# Integrated Metabolomics and Transcriptomics Reveal the Key Role of Flavonoids in the Cold Tolerance of Chrysanthemum

**DOI:** 10.3390/ijms25147589

**Published:** 2024-07-10

**Authors:** Di Wu, Yingxue Wu, Ruiqi Gao, Yanhong Zhang, Ruiying Zheng, Minghui Fang, Yuhua Li, Yang Zhang, Le Guan, Yanqiang Gao

**Affiliations:** 1 Key Laboratory of Saline-Alkali Vegetation Ecology Restoration, Ministry of Education, Northeast Forestry University, Harbin 150040, China; wd0809@nefu.edu.cn (D.W.); 121945gao@nefu.edu.cn (R.G.);; 2 College of Life Science, Northeast Forestry University, Harbin 150040, China

**Keywords:** Chrysanthemum, low temperature, metabolome, transcriptome, flavonoids, anthocyanins

## Abstract

Chrysanthemum (*Chrysanthemum morifolium*, ground-cover Chrysanthemums), one of the important garden flowers, has a high ornamental and economic value. However, its ornamental value is significantly diminished by the low temperature experienced in northeastern China. Here, metabolomics and transcriptomics were performed on three Chrysanthemum cultivars before and after a low temperature to investigate the dynamic metabolite changes and the molecular regulatory mechanisms. The results showed that 1324 annotated metabolites were detected, among which 327 were identified as flavonoids derived from Chrysanthemum. The accumulation of metabolites and gene expression related to the flavonoid biosynthesis pathway significantly increased in the three cultivars under the low temperature, indicating flavonoid metabolism actively participates in the Chrysanthemum cold response. Specifically, the content of cyanidin and pelargonidin derivatives and the expression of anthocyanin biosynthesis genes significantly increases in XHBF, providing a reasonable explanation for the change in petal color from white to purple under the low temperature. Six candidate UDP-glycosyltransferase genes involved in the glycosylation of flavonoids were identified through correlation networks and phylogenetic analysis. CmNAC1, CmbZIP3, and other transcription factors potentially regulating flavonoid metabolism and responding to low temperatures were discovered by correlation analysis and weighted gene co-expression network analysis (WGCNA). In conclusion, this study elucidated the specific response of flavonoids to low temperatures in Chrysanthemums, providing valuable insights and metabolic data for investigating cold tolerance.

## 1. Introduction

Chrysanthemum originated in China and has been cultivated for over three thousand years. As one of the four major cut flowers, the Chrysanthemum is highly favored by people and possesses a significant market demand [1,2,3]. Due to its exceptional ornamental and economic value, Chrysanthemum has been extensively grown worldwide as a crucial economic flower crop. Furthermore, Chrysanthemum is widely utilized in the medical, food, and beverage industries owing to its nutritional value and bioactive components [4].

Cold stress, including chilling (<15 °C) and/or freezing (<0 °C) temperatures, adversely affects plant growth and development and limits their spatial distribution and agricultural productivity [5]. When plants are exposed to low temperatures, various changes occur such as changes in the cell membrane structure, chlorophyll content, and stomatal conductance [6]. Additionally, photosynthesis is highly sensitive to temperature change. Under the low temperature, the plastoquinone levels will decrease, which leads to photo-inhibitory damage to photosynthetic system II (PS II). Furthermore, low temperatures result in the accumulation of osmotic substances (proline and soluble sugars) in plants, which contribute to the osmoregulation of plants under cold stress. Moreover, the permeability of cell membranes, reactive oxygen species (ROS), and plant hormones undergo varying degrees of change [7,8]. In recent decades, the cold signaling pathway focused on CBF (C-repeat-binding factors) transcription factors has been extensively researched and gradually elucidated. *Arabidopsis thaliana* possesses three *CBF* genes, namely *CBF1* (*DREB1B*), *CBF2* (*DREB1C*), and *CBF3* (*DREB1A*), which can bind to a conserved CRT/DRE (C-repeat/dehydration response element) regulatory element CCGAC in the promoter region of *COR* (*Cold-regulated*) gene in response to low temperature [9,10,11,12]. The overexpression of *CBF1*-*CBF*3 significantly enhances cold tolerance in *Arabidopsis*, leading to the constitutive induction of large numbers of *COR* genes [10,13]. Furthermore, it is worth noting that *CBF* is also regulated by other transcription factors, with ICE1 (inducer of CBF expression 1) serving as a positive regulator of CBF. Notably, compared with the wild type, the cold induction of the *CBF3* gene in the *ice1* mutant was severely inhibited, resulting in significantly reduced cold tolerance [14,15]. However, several studies have demonstrated that some cold response genes are *CBF*-independent in plants [16,17,18,19]. Since cold stress is a common stress factor that significantly hinders Chrysanthemum’s productivity and economic value, it is crucial to study the response of Chrysanthemums to cold stress.

Flavonoids, as a type of polyphenols, are important natural products widely distributed in plants, characterized by a C6-C3-C6 benzene ring structure. It is formed by connecting two benzene rings (A and B rings) and a three-carbon chain, which in many flavonoids further forms a heteropyran or pyrone ring (C ring) [20,21]. They play a crucial role in plant growth and development, including resistance to biotic and abiotic stresses, the regulation of auxin transport and metabolism, and the promotion of plant symbiosis with fungi, as well as influencing plant fertilization and reproduction [22,23]. As part of cellular antioxidant mechanisms, flavonoids reduce free radical formation and scavenge stress-induced ROS to prevent destructive protein oxidation, lipid peroxidation, and nucleic acid rupture in stressed cells. The structure and substituents in B and C rings mediate the antioxidant activity [20,24]. The presence of positively charged oxygen atoms in the C ring makes anthocyanins the potent antioxidants among flavonoids. In addition, considering nutritional and health care, flavonoids have important roles such as antioxidant, anti-tumor, anti-inflammatory, anti-malaria, etc. Among them, anthocyanins and proanthocyanidins are used in the market as food preservatives and colorants [25].

The biosynthesis of flavonoids primarily begins with phenylalanine, and then p-coumaroyl-CoA is synthesized through the action of phenylalanine ammonia-lyase (PAL), cinnamic acid 4-hydroxylase (C4H), and 4-coumarate: CoA ligase (4CL). Following this, one p-coumaroyl-CoA and three malonyl-CoA form naringin chalcone under the action of chalcone synthetase (CHS), resulting in the formation of two benzene rings. The formation of the C ring is catalyzed by chalcone isomerase (CHI) to produce naringin. Naringin can produce dihydrokaempferol through flavanone 3 hydroxylase (F3H). Then dihydrokaempferol can produce proanthocyanidins by the catalytic action of dihydroflavonol-4-reductase (DFR). Subsequently, pelargonidin and cyanidin are produced by the action of anthocyanin synthase (ANS). Flavonoid synthetase (FNS) can catalyze the conversion of naringin into apigenin, which is further transformed into luteolin through the action of flavonoid 3′-hydroxylase (F3′H). Numerous studies have demonstrated that transcription factors (TFs) regulate flavonoid biosynthesis. It is widely accepted that the MBW (MYB-bHLH-WD40) complexes play a crucial role in regulating the structural genes involved in flavonoid biosynthesis. In roses, two bHLH (RcbHLH42 and RcEGL1) bind to RcMYB1 and RcTTG1 to form MBW complexes that regulate anthocyanin biosynthesis [26]. The interaction between MdMYB308L and MdbHLH33 in apples positively regulates cold tolerance and anthocyanin biosynthesis [27]. Additionally, TFs responding to abiotic stress by regulating flavonoid biosynthesis have also been reported in Chrysanthemums. For instance, in Chrysanthemum, high temperatures induce *CmMYB012* which directly regulates CmFNS to inhibit flavonoid biosynthesis [28]. DgMYB2 directly binds to the promoter of DgGPX1 and increases glutathione peroxidase (GPX) activity to reduce the accumulation of ROS, thereby enhancing the Chrysanthemum’s cold tolerance [29]. The demethylation of CmMYB6 increases anthocyanin content in Chrysanthemums, affecting their color [30]. Although there were several reports on cold tolerance in Chrysanthemums, research on flavonoid metabolism and glycosylated flavonoid response to cold still needs to be completed.

Here, we selected three Chrysanthemum cultivars with different performances under a low temperature for metabolome and transcriptome analysis to elucidate the metabolite accumulation and gene expression changes of the flavonoid biosynthesis pathway under a low temperature in the Chrysanthemum. At the same time, glycosyltransferase genes in Chrysanthemum were excavated, and their potential products were analyzed. In addition, this study constructed a correlation network between flavonoid biosynthesis pathway genes and transcription factors under a low temperature in Chrysanthemums and identified a potential cold-tolerant transcription factor.

## 2. Results

### 2.1. Phenotypic Changes under the Low Temperature

**Figure 1:**
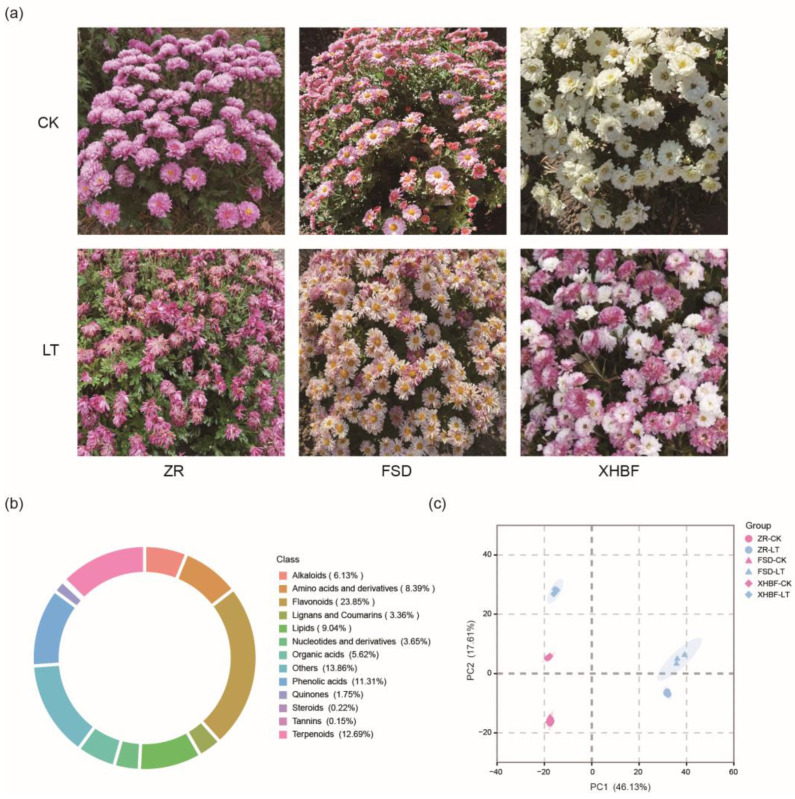
Phenotypes and metabolite profiles of ZR, FSD, and XHBF under CK and LT. (**a**) Phenotypic differences of ZR, FSD, and XHBF between CK and LT. The upper half of the picture represents CK, and the lower half represents LT. (**b**) The classification of metabolites detected in the three Chrysanthemum cultivars. (**c**) Principal component analysis of metabolites under CK and LT in the three Chrysanthemum cultivars. PC1 represents the first principal component, and PC2 represents the second principal component.

Three Chrysanthemum cultivars collected under the normal temperature (CK) and the low temperature (LT; refer to Materials and Methods for details) were selected for this study to investigate the effects of the low temperature on Chrysanthemums. Phenotypic observations revealed that the three cultivars exhibited different changes under the low temperature (Figure 1a). ZR showed the most significant change, with its petals displaying significant atrophy and wilting. In contrast, this phenomenon did not appear in FSD and XHBF. It was worth noting that the color transition from white to purple in XHBF under the low temperature suggested a possible change in anthocyanin accumulation.

### 2.2. Metabolite Profile under the Low Temperature of Chrysanthemum Petal

To further explore the dynamic changes of metabolites under the low temperature, metabolome analysis was performed based on a widely targeted LC-MS/MS (liquid chromatography–tandem mass spectrometry). A total of 1371 annotated metabolites were detected, including 84 alkaloids, 115 amino acids and derivatives, 327 flavonoids, 46 lignans and coumarins, 124 lipids, 50 nucleotides and derivatives, 77 organic acids, 115 phenolic acids, 24 quinines, 174 terpenoids, three steroids, 12 tannins, and 190 other metabolites (Figure 1b). Principal component analysis (PCA) was conducted to compare the variations in metabolite accumulation responding to low temperature among the three cultivars. Significant differences were found in the first principal component (PC1) between ZR-CK and ZR-LT, as well as between FSD-CK and FSD-LT, with a variation contribution rate of 46.47%. The separation between XHBF-CK and XHBF-LT was observed in the second principal component (PC2), with a variation contribution rate of 17.02%. Furthermore, separations were still evident between ZR-CK and ZR-LT and between FSD-CK and FSD-LT in PC2 (Figure 1c).

**Figure 2:**
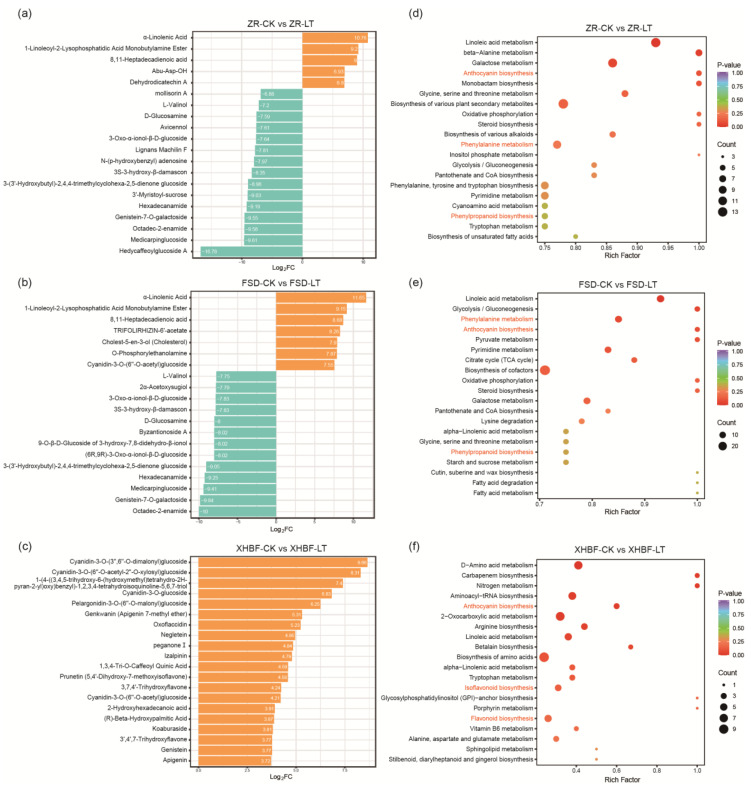
Analysis of differently accumulated metabolites (DAMs) in the three comparison groups (ZR-CK vs. ZR-LT, FSD-CK vs. FSD-LT, and XHBF-CK vs. XHBF-LT). (**a**–**c**) The top 20 metabolites with the highest foldchange number in the three comparison groups. (**d**–**f**) KEGG enrichment analysis of DAMs in three comparison groups. The red font represents the pathways of focus.

We further identified differential accumulation metabolites (DAMs) based on |Log_2_FC| ≥ 1 and VIP > 1 values to explore which metabolites were significantly changed under the low temperature. Volcano plots showed that 741 (479 up-regulated, 262 down-regulated), 789 (508 up-regulated, 281 down-regulated), and 217 (187 up-regulated, 30 down-regulated) DAMs were detected in ZR-CK vs. ZR-LT, FSD-CK vs. FSD-LT, and XHBF-CK vs. XHBF-LT, respectively (Supplementary Figure S1). Surprisingly, the majority of the metabolites with the top 20 difference ratios in ZR and FSD were down-regulated DAMs, whereas all top 20 metabolites were up-regulated DAMs in XHBF (Figure 2a–c). Of particular note was the presence of 15 flavonoids in XHBF, including apigenin, genistein, cyanidin-3-O-glucoside, cyanidin-3-O-(6″-O-acetyl) glucoside, cyanidin-3-O-(3″,6″-O-dimalonyl) glucoside, and pelargonidin-3-O-(6″-O-malonyl) glucoside, etc. The accumulation of cyanidin and pelargonidin derivatives played crucial roles in influencing the appearance of purple and red Chrysanthemums. These results elucidated why XHBF exhibited a color shift under the low temperature. We also observed that α-linolenic acid was significantly increased in ZR and FSD under the low temperature, and L-Valinol was significantly decreased in both varieties. Interestingly, these changes were not present in XHBF.

Then, the DAMs from the three comparison groups were assigned to the Kyoto Encyclopedia of Genes and Genomes (KEGG) to investigate the metabolic pathway changes under the low temperature (Figure 2d–f). The results revealed that linolenic acid metabolism and anthocyanin biosynthesis were all enriched in the three comparison groups. Phenylpropanoid biosynthesis and phenylalanine metabolism were enriched in ZR and FSD, while flavonoid biosynthesis and isoflavone biosynthesis were enriched in XHBF. Notably, phenylalanine metabolism was a crucial upstream pathway for flavonoid biosynthesis and anthocyanin biosynthesis. These results indicated the significant role of flavonoids in Chrysanthemum’s cold tolerance. Additionally, glycolysis/gluconeogenesis and the citric acid cycle exhibited enrichment, suggesting sugar and lipid metabolism changed under the low temperature.

### 2.3. Transcriptome Analysis of the Three Chrysanthemum Cultivars Responding to Low Temperature

RNA libraries were constructed and sequenced using the Illumina platform to investigate the gene expression changes of the three cultivars under the low temperature. After filtering the raw data and assessing sequencing error rates and GC content distribution, approximately 208.45 Gb of clean data was obtained from the 18 cDNA libraries (three replicates per sample). Each library ranged from 4.81–5.27 GB with a percentage of Q30 bases ≥ 92%.

**Figure 3:**
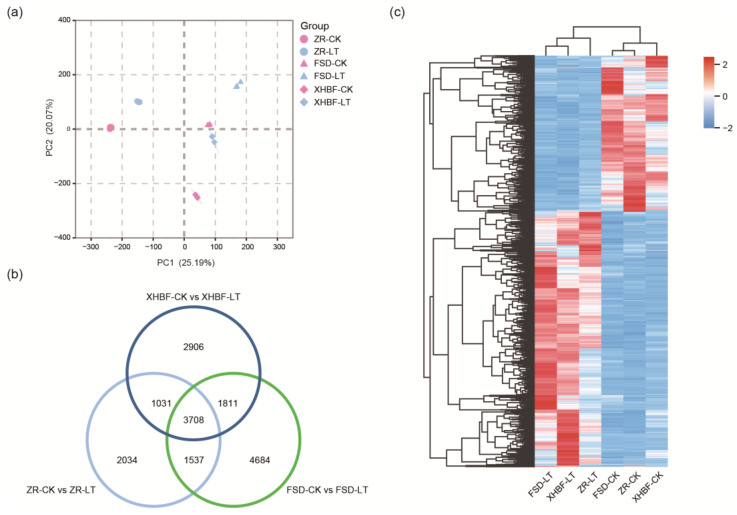
Transcriptome analysis of ZR, FSD, and XHBF under CK and LT. (**a**) Principal component analysis. (**b**) Venn diagram showing differentially expressed genes (DEGs) in the three comparison groups. (**c**) Expression heatmap of overlapped DEGs in the three comparison groups.

The PCA based on fragments per kilobase of exon model per million mapped fragments (FPKM) revealed significant differences in PC1 and PC2 between ZR-CT and ZR-LT, and between FSD-CK and FSD-LT, as well as between XHBF-CK and XHBF-LT. The variance contribution of PC1 was 25.19%, while the variance contribution of PC2 was 20.07% (Figure 3a). Differentially expressed genes (DEGs) were identified based on |Log_2_FC| ≥ 1 and false discovery rate (FDR < 0.05). A total of 8310 (3998 up-regulated, 4312 down-regulated), 11,740 (5199 up-regulated, 4257 down-regulated), and 9456 (3998 up-regulated, 4312 down-regulated) genes were identified in ZR-CK vs. ZR-LT, FSD-CK vs. FSD-LT, and XHBF-CK vs. XHBF-LT, respectively (Supplementary Figure S2). There were 3708 genes that overlapped in the three comparison groups (Figure 3b). The different temperature conditions (CK and LT) could be completely separated according to the expression pattern of the overlapped genes. Interestingly, the expression patterns were more similar for FSD and ZR under CK but closer for FSD and XHBF under LT. Furthermore, most of these genes increased their expression under LT (Figure 3c).

To explore the potential biological significance of these DEGs, KEGG and gene ontology (GO) were employed. The KEGG results revealed that fatty acid elongation, zeaxanthin biosynthesis, carotenoid biosynthesis, brassinosteroid biosynthesis, and phenylpropane biosynthesis were significantly enriched in the three comparison groups. Flavonoid biosynthesis was enriched in FSD-CK vs. FSD-LT and XHBF-CK vs. XHBF-LT, while isoflavone biosynthesis was specifically enriched in XHBF-CK vs. XHBF-LT. Additionally, sucrose and starch metabolism were also found to be significantly enriched in ZR-CK vs. ZR-LT and XHBF-CK vs. XHBF-LT (Supplementary Figure S3). These findings suggested that flavonoids, sugars, lipids, and carotenoids might be involved in the low-temperature response in Chrysanthemums. GO functional analysis showed that flavonoid metabolism was enriched in the biological process among the three comparison groups (Supplementary Figure S4). UDP-glycosyltransferase activity exhibited the highest enrichment in the three comparison groups’ molecular functional modules. This indicated its potential role in response to the low temperature. Flavonoids typically exist in plants as glycosides, with glycosylation playing a crucial role in pigment stability, solubility, and cold tolerance. The alterations observed here suggested that flavonoid metabolism might be pivotal to the low-temperature response in Chrysanthemums.

### 2.4. Transcript and Metabolite Changes in Flavonoid Biosynthesis Pathways under the Low Temperature

**Figure 4:**
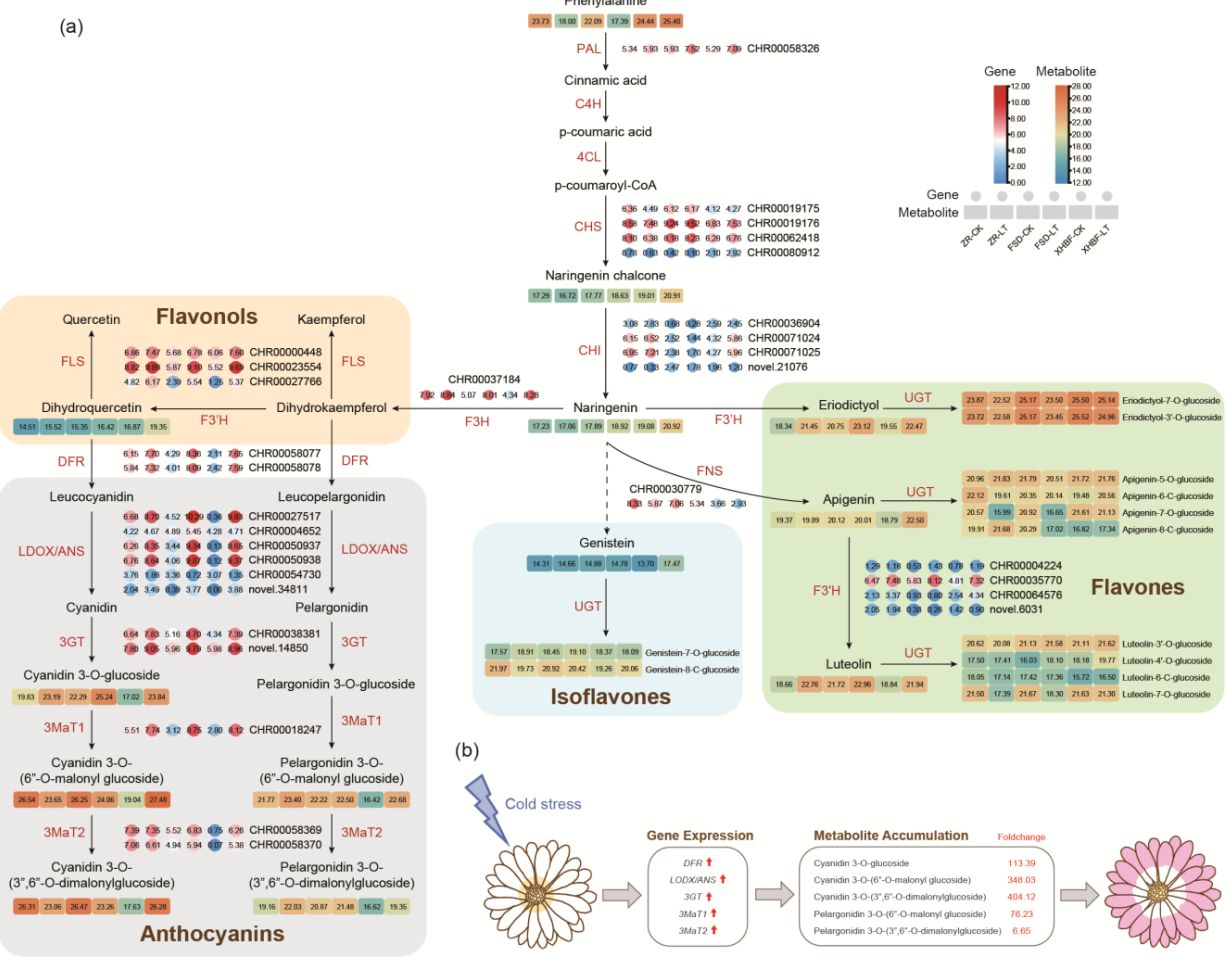
Flavonoid biosynthesis pathway and a hypothetical model of XHBF color alternation. (**a**) Gene expression and metabolite accumulation in phenylalanine metabolism and flavonoid biosynthesis pathways. The abundance of metabolites and the FPKM values of genes were transformed using a logarithmic scale. PAL, phenylalanine ammonia-lyase; C4H, cinnamate 4-hydroxylase; 4CL, 4-coumarate: CoA ligase; CHS, chalcone synthase; CHI, chalcone isomerase; F3H, flavanone 3-hydroxylase; F3′H, flavonoid 3′-hydroxylase; DFR, dihydroflavonol 4-reductase; LDOX/ANS, Leucoanthocyanidin dioxygenase/anthocyanidin synthase; 3GT, UDP-glucose: anthocyanidin 3-glucosyltransferase; 3MaT1, anthocyanin 3-O-glucoside-6″-O-malonyltransferase; 3MaT2, anthocyanidin 3-O-glucoside-3″,6″-O-dimalonyltransferase; FNS, flavone synthase; FLS, flavonol synthase; UGT, UDP-glycosyltransferase. (**b**) A hypothetical model of XHBF color transformation. The red arrow represents that gene expression was increased. The red numbers represent the foldchange between CK vs. LT in XHBF.

Given the enrichment in the flavonoid pathway under the low temperature in the three cultivars, the transcript and metabolite changes in the pathway were indicated in Figure 4a. The accumulation of phenylalanine in ZR and XHBF was higher than in FSD under CK. When considering the temperature conditions, its accumulation in ZR and FSD was significantly reduced under LT, while it slightly increased in XHBF. The transcript of *PAL* (*CHR00058326*) showed a significant increase in FSD and XHBF but exhibited no difference in ZR. The expression of *CHS* genes (*CHR00019175*, *CHR00019176*, and *CHR00062418*) was similar to *CHR00058326*. The low temperature increased their expression in FSD and XHBF while decreasing their expression in ZR. The content of naringin chalcone increased in FSD and XHBF but slightly decreased in ZR under LT, which was consistent with the gene expression of *CHS*. Naringenin was produced from naringenin chalcone through the catalyzation of CHI. Two *CHI* genes, *CHR00071024* and *CHR00071025*, highly expressed in ZR and XHBF, were upregulated under LT, especially in XHBF, more than three times compared to CK. Although there were differences in gene expression among the three cultivars, the accumulation pattern observed for naringenin was consistent with naringenin chalcone. This phenomenon was closely associated with their performance under the low temperature.

Naringin produces dihydrokaempferol through the action of F3H. We identified a gene (*CHR000037184*) encoding F3H. Its expression increased from low to high in ZR, FSD, and XHBF. *CHR00030779*, encoding FNS, decreased by approximately six, four, and two times in ZR, FSD, and XHBF, respectively. However, the accumulation of apigenin showed no significant change in ZR and FSD but exhibited an increase in XHBF. This suggested that there may be other regulators of flavonoid accumulation. Four genes (*CHR00004224*, *CHR00035770*, *CHR00064576*, and *novel.6031*) encoding F3′H were identified, and only *CHR00035770* showed high expression. We found that the expression of *CHR00035770* was, respectively, increased about two, five, and six folds in ZR, FSD, and XHBF, which was consistent with the accumulation of eriodictyol, dihydroquercetin, and luteolin. Additionally, the expression of the three genes (*CHR00000448, CHR00023554,* and *CHR00027766*) encoding FLS was notably upregulated by low-temperature induction.

Isoflavones have not been extensively studied in Chrysanthemum, but we detected some isoflavones, and the significance of this should not be underestimated. Genistein accumulation showed no difference in ZR and FSD but increased sharply in XHBF. The accumulation of genistein 7-O-glucoside and genistein 8-C-glucoside exhibited an opposite trend responding to the low temperature in the three cultivars. Genistein 7-O-glucoside increased in ZR and FSD and decreased in XHBF under LT; genistein 8-C-glucoside decreased in ZR and FSD and increased in XHBF.

### 2.5. Transcript and Metabolite Changes in Anthocyanin Biosynthesis Pathways under the Low Temperature

The accumulation of anthocyanins has been shown to enhance the cold tolerance of plants. The expression of *DFR* (*CHR00058077* and *CHR00058078*) was significantly upregulated in the three cultivars under LT. Specifically, the expression of these two genes in ZR was approximately three times higher than CK, and it increased about 18 times in FSD. Notably, the expression of these two genes in XHBF under LT was 59 and 44 times higher than under CK, respectively. The expression of *CHR00027517*, encoding LDOX, was upregulated by about four-fold under LT in ZR and approximately sixty-fold in FSD. Interestingly, the FPKM value for XHBF approached 0 under CK but rose to 908 under LT. Similarly, the genes *CHR00004652*, *CHR00050937*, and *CHR00050938* encoding ANS also exhibited similar expression patterns. We observed a differential accumulation of cyanidin-3-O-(6″-O-malonyl) glucoside and cyanidin-3-O-(3″,6″-O-dimalonyl) glucoside among the three cultivars under LT. Additionally, there was an increase in pelargonidin-3-O-(6″-O-malonyl) glucoside and pelargonidin-3-O-(3″,6″-O-dimalonyl) glucoside accumulation among the three cultivars. We observed an increase in the expression of two genes encoding 3GT (*CHR00038381*, *novel.14850*) in all three cultivars. Similarly, the expression of 3MAT1 (*CHR00018247*) also showed an increase in the three cultivars. However, the expression of *3MAT2* (*CHR00058369* and *CHR00058370*) decreased in ZR but increased in FSD and XHBF. These findings suggested that there were variations in anthocyanin accumulation among the three Chrysanthemum cultivars under LT. Additionally, we proposed a hypothetical model for the phenotypic changes in XHBF (Figure 4b). Genes related to anthocyanin biosynthesis in XHBF exhibited significant upregulation under LT, with some genes showing a transition from no expression to high expression. This led to an increased accumulation of cyanidin and pelargonidin derivatives within the petals, resulting in a color change from white to purple for XHBF.

### 2.6. The Expression of UDP-Glycosyltransferase Changed under the Low Temperature

**Figure 5:**
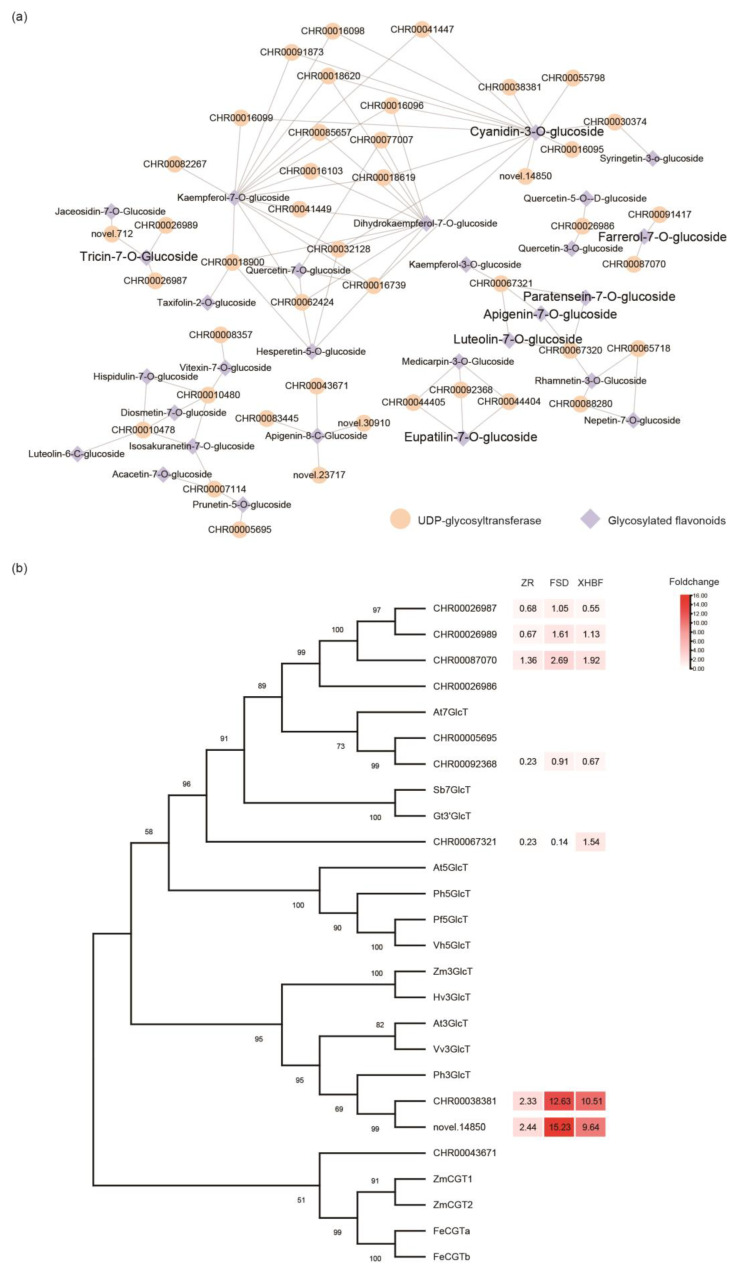
Correlation analysis between UDP-glycosyltransferase and glycosylated flavonoids and phylogenetic analysis. (**a**) Correlation network of UDP-glycosyltransferase and glycosylated flavonoids. (**b**) Phylogenetic tree of UDP-glycosyltransferase in Chrysanthemum and flavonoid glycosyltransferases in other species.

The glycosylation of flavonoids is catalyzed by UDP-glycosyltransferases (UGTs). The UGT family genes have undergone continuous expansion, differentiation, and specialization during plant evolution. This has led to the formation of UGT gene families with different substrate and region specificity, synthesizing flavonoid glycosides with diverse components and functions in various plants [31]. Here, we identified 88 genes annotated as UGT through the Non-Redundant (NR) Protein Database. The potential regulatory relationship between these genes and glycosylated flavonoids was investigated by correlation analysis (only positive correlation was considered, r > 0.8). The results revealed significant correlations between 45 genes and 34 glycosylated flavonoids (Figure 5a). Additionally, phylogenetic trees were constructed for the 45 genes and 21 reported flavonoid UGTs. *CHR00038381* has been characterized as anthocyanin 3-O-glycosyltransferase, significantly positively correlated with cyanidian-3-O-glucoside. This illustrated the reliability of our correlation network. *Novel.14850* was a new transcript annotated as anthocyanin 3-O-glycosyltransferase in the NR database and related to cyanidin 3-O-glucoside. *CHR00067321* was significantly correlated with apigenin-7-O-glucoside, luteosin-7-O-glucoside, and paratensein-7-O-glucoside. Tricin-7-O-glucoside was related to *CHR00026987* and *CHR00026989*. Eupatilin-7-O-glucoside and farrerol-7-O-glucoside were associated with *CHR00092368* and *CHR00087070*, respectively. The phylogenetic tree showed that *CHR00067321*, *CHR00026987*, *CHR00026989*, *CHR00092368*, and *CHR00087070* were highly homologous to *At7GlcT* and *Sb7GlcT* (Figure 5b). These findings provided potential options for exploring flavonoid glycosylation modifications in Chrysanthemum.

### 2.7. Regulatory Network of Transcription Factors and Flavonoid Biosynthesis Genes

**Figure 6:**
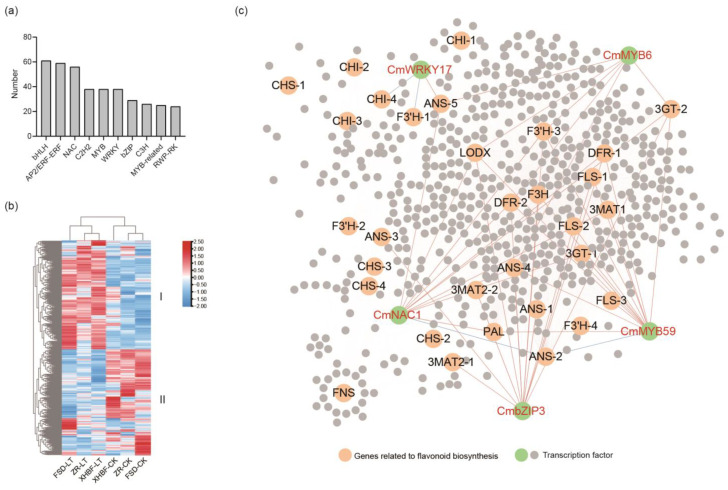
The screen of candidate transcription factors related to low-temperature response in the three Chrysanthemum cultivars. (**a**) Top ten TF families with the highest number of differentially expressed genes. (**b**) Expression heat map of differentially expressed TFs. All values are log-normalized and row-normalized. I and II represent different clusters. (**c**) Correlation network of TFs and genes related to phenylalanine metabolism, flavonoid biosynthesis pathways. The red line represents a positive correlation, and the blue line represents a negative correlation. Different numbers represent different coding genes for the same enzyme, as detailed in Table S1.

Transcription factors (TFs) play a crucial role in regulating flavonoid metabolism. In this study, 2474 transcriptions were identified, with 695 showing differential expression between CK and LT. The bHLH family was the most numerous, consisting of 61 genes, followed by 59 genes in the AP2/ERF-ERF family and 56 genes in the NAC family (Figure 6a). We mapped the expression heatmap of these TFs, which revealed that CK and LT could be clearly distinguished into two groups through longitudinal clustering in the three cultivars. Horizontal clustering categorized these TFs into two clusters. Cluster I represented TFs with up-regulated expression under the low temperature, while cluster II represented TFs with down-regulated expression (Figure 6b).

To further investigate the relationship between these TFs and flavonoid biosynthesis pathway genes, we constructed a correlation network based on r > 0.7 (Figure 6c). Previous studies have reported that cold stress at 4 °C could induce the expression of *CmMYB59*. We observed a significant positive correlation between *CmMYB59* and several genes, including *PAL*, *LDOX*, *DFR-1*, and *DFR-2*, in the correlation network. *CmMYB6* has been shown to activate DFR and thus promote anthocyanin accumulation. Our findings also show a significant positive correlation between *CmMYB6* and *DFR-2*. Additionally, we found that *CmMYB6* was positively correlated with *LDOX*, *ANS-4*, and *ANS-5*, indicating its potential role in regulating the transition process of proanthocyanidins to anthocyanins for anthocyanin accumulation. The expression of *CmWRKY17* was induced by salt stress. Here, we found that its expression was also altered under LT. The analysis indicated a positive correlation with *ANS-5* and a negative correlation with *CHI-4* and *F3′H-1*. Furthermore, we identified a NAC transcription factor called CmNAC1, which was significantly induced by low-temperature stress. Its expression was extremely high in all three cultivated species and approximately four-fold increased under LT. The correlation network showed that it was significantly positively correlated with most genes involved in the flavonoid synthesis pathway. Interestingly, we found that *CmNAC1* and *CmMYB59* were significantly negatively related to *ANS-2*, although the expression of *ANS-2* was very low.

Notably, we identified a bZIP gene named *CmbZIP3* (*CHR000050023*) showing a significant positive correlation with anthocyanin biosynthesis genes, such as *LDOX*, *ANS-1*, *ANS-3*, *ANS-4*, *DFR-1*, *DFR-2*, *3MAT2-1*, *3MAT2-2*, and so on. This suggested that *CmbZIP3* might impact the cold tolerance of Chrysanthemum by regulating anthocyanin biosynthesis. Of particular interest was our discovery that previous studies have reported a transcription factor, DgbZIP3, which regulates cold tolerance in Chrysanthemum. The overexpression of *DgbZIP3* significantly improves cold tolerance and it can also interact with DgbZIP2 to regulate Chrysanthemum cold tolerance [32]. Through protein sequence comparison, we found that *CHR000050023* shared the same conserved domain (NRESARRSR) as DgbZIP3 (Supplementary Figure S5). This indicated that *CmbZIP3* might also possess the ability to enhance the cold tolerance of Chrysanthemums.

### 2.8. Weighted Gene Co-Expression Network Analysis

**Figure 7:**
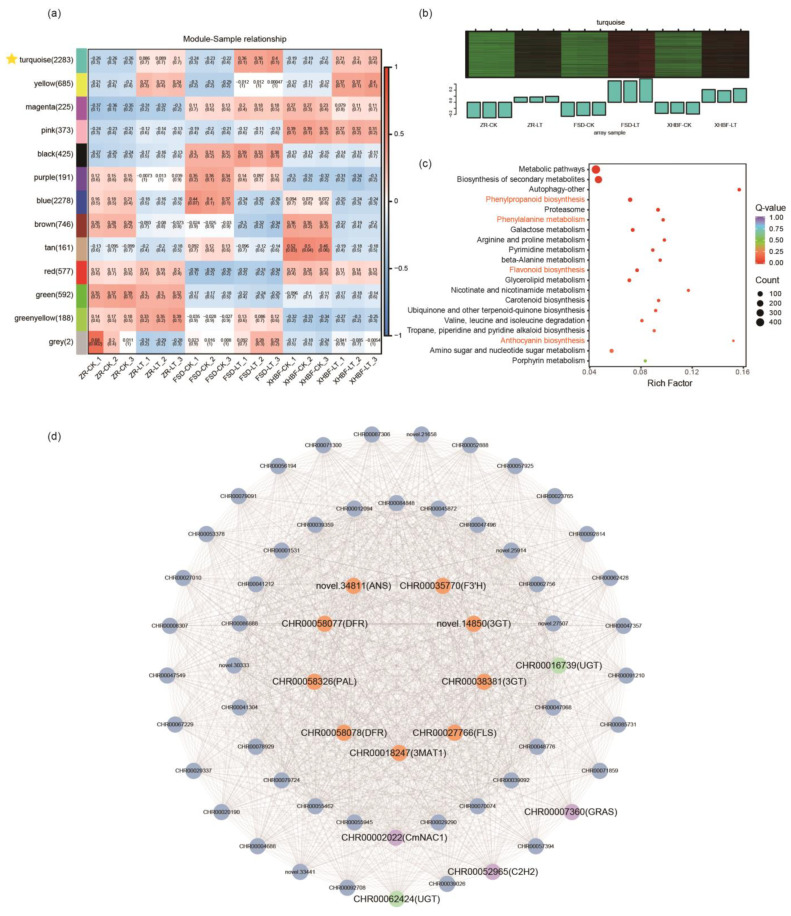
Weighted gene co-expression network analysis. (**a**) Module-sample relationships. Each cell contains the corresponding correlation and *p*-value. Red and blue represent positive and negative correlations, respectively, and the darker the color, the stronger the correlation. (**b**) Gene expression pattern of the MEturquoise module. (**c**) KEGG enrichment analysis of genes in MEturquoise. The red font represents the pathway of focus. (**d**) Co-expression network of flavonoid biosynthesis genes in MEturquoise.

Weighted gene co-expression network analysis was utilized to investigate gene clusters associated with the low-temperature response in Chrysanthemums. By constructing a systematic cluster tree and heat map to identify the relevant feature gene set, 8848 genes were categorized into 16 modules (Supplementary Figure S6). The MEturquoise module was particularly concerned due to its negative correlation with the three cultivars under CK, and its positive correlation under the low temperature (Figure 7a). The heatmap of the module revealed that gene expression in the MEturquoise module of the three Chrysanthemum cultivars was lower under CK than LT (Figure 7b).

To comprehensively understand the genes in the MEturquoise module, we conducted a KEGG enrichment analysis on these genes. The analysis results indicated significant enrichment of flavonoid biosynthesis and anthocyanin biosynthesis pathways within this module (Figure 7c,d). After further investigation, we identified seven flavonoid biosynthesis genes that were previously mapped and two UGTs in this module. Subsequently, the genes linked to these nine genes were selected, and the top 50 genes with connectivity were screened to construct a gene co-expression network. The findings revealed that three transcription factors (NAC, C2H2, and GARS) were connected to all of the genes in the network. This suggested that these three transcription factors were significantly induced by the low temperature and potentially regulated with other genes in the network. Notably, the NAC transcription factor was the previously mentioned CmNAC1, which further confirms that it might regulate the expression of the flavonoid biosynthesis pathway genes.

## 3. Discussion

The secondary metabolites of plants play a crucial role in ensuring the organism’s viability within its environment. Abiotic stresses will disturb plant metabolism, so it must reprogram its metabolic network to adapt to the unfriendly growth environment. The emergence of metabolomics provides a powerful tool for comprehensively understanding the changes in plant metabolic networks under stress [33,34]. Here, 1231 annotated metabolites were detected in three Chrysanthemum cultivars (ZR, FSD, and XHBF) using UHPLC-MS, encompassing both primary and secondary metabolites. Notably, the largest proportion among these metabolites was occupied by 321 flavonoids. This provides a valuable resource for investigating the mechanism of Chrysanthemum low-temperature response. PCA revealed significant changes in the metabolite profiles of all three cultivars under the low temperature. Furthermore, an increase in anthocyanins such as cyanidin-3-O-glucoside, cyanidin-3-O-(6″-O-acetyl) glucoside, cyanidin-3-O-(3″,6″-O-dimalonyl) glucoside, and pelargonidin-3-O-(6″-O-malonyl) glucoside was explicitly noted in XHBF, which explained the color transition from white to purplish red observed in this particular variety.

As important secondary metabolites, flavonoids are crucial in protecting plants against abiotic stresses such as UV-B radiation, salinity, drought, and extreme temperatures [20,21,24,35]. The removal of ROS is an essential part of plants’ response to abiotic stress. Due to their unique structure, flavonoids possess potent antioxidant properties and can effectively eliminate ROS while preventing their generation. Previous studies have indicated a close relationship between the expression of the anthocyanin synthase (BrANS) gene and cold stress tolerance [36]. Specifically, the deletion of anthocyanin pigment 1 (PAP1) MYB transcription factor has been found to impair cold tolerance in *Arabidopsis* leaves [37]. Research in apple and *Liriope spicata* has shown that genes and metabolites on the flavonoid pathway can participate in osmoregulation during freezing stress [38,39]. KEGG analysis of genes and metabolites revealed a significant enrichment of flavonoid biosynthesis and anthocyanin biosynthesis in ZR-CK vs. ZR-LT, FSD-CK vs. FSD-LT, and XHBF-CK vs. XHBF-LT. An in-depth analysis of the flavonoid biosynthesis pathway revealed that the accumulation of flavonoids in three Chrysanthemum cultivars tended to increase under the low temperature. XHBF showed a significant increase compared to ZR and FSD in terms of flavonoid accumulation and gene expression. This suggested that XHBF could better withstand the cold due to its response to the low temperature. Additionally, we observed significant changes in anthocyanin accumulation among all three Chrysanthemum cultivars. The essential genes controlling anthocyanin biosynthesis, *DFR* and *LDOX/ANS*, significantly increased, especially in XHBF. Interestingly, there were differences in cyanidin and pelargonidin accumulation under the low temperature among the different Chrysanthemum cultivars. The substantial increase in cyanidin and pelargonidin derivatives in XHBF confirms its discoloration ability.

Flavonoids are mainly found in plants in the form of glucosides and play a crucial role in maintaining REDOX homeostasis and conferring abiotic stress tolerance. Glycosylation usually occurs in the final step of flavonoid biosynthesis, and this modification can enhance the solubility, stability, bioactivity, and diversity of the compound. The study found that various abiotic stimuli, such as cold, salt, and drought, significantly enhanced the expression of UGTs [40,41,42,43]. In *Arabidopsis thaliana*, two stress-induced anthocyanin rhamnosyl transferases (UGT79B2 and UGT79B3) confer plant adaptations to cold, salt, and drought stress by regulating anthocyanin metabolism [44]. In tea plants, three UGTs (CsUGT91Q2, CsUGT78A14, and CsUGT78A15) increase cold tolerance through glycosylation of sesquiterpenes and flavonols [45]. However, the research on flavonoid glycosyltransferase in Chrysanthemum is not clear. Here, we identified 88 genes encoding UGTs, and the expression of most genes increased under the low temperature. This suggested that UGTs in Chrysanthemums were also actively involved in low-temperature response. Although they belong to the same family, different UGTs act on various substrates and modify locations. Based on the phylogenetic tree and correlation analysis, we identified one anthocyanin 3-O-glucoside transferase and five flavonoid 7-O-glucoside transferases. This provided a basis for a further study of glycosyltransferase’s function in Chrysanthemum.

It has been widely reported that transcription factors enhance plant resilience by regulating flavonoid biosynthesis. The NAC transcription factor family was found to have the largest number of differentially expressed transcription factors. GmNAC20 regulates COR and mediates stress tolerance by directly binding the promoter of *DREB1A* and inhibiting the expression of *DREB1C* [46]. In apples, MdNAC104 can positively regulate cold tolerance by promoting anthocyanin accumulation through CBF-dependent and CBF-independent pathways [47]. In addition, MBW complexes have been reported to affect cold tolerance by regulating anthocyanins in *Arabidopsis*, rose, and apple [26,27,48]. Based on correlation networks, we explored the potential regulatory relationship between transcription factors and flavonoid biosynthesis pathway genes. CmMYB6 and CmMYB59 have been demonstrated to regulate the expression of anthocyanin biosynthesis genes in response to low temperatures. We found that they were significantly positively correlated with flavonoid and anthocyanin synthesis genes. We also identified a bZIP transcription factor CmbZIP3 with the same conserved motif as DgbZIP3. Previous studies have shown that the overexpression of *DgbZIP3* could enhance cold resistance by increasing the activity of peroxidase (POD) in Chrysanthemum, while antisense inhibition reduced the activity of POD, resulting in weaker cold resistance [32]. The expression of *CmbZIP3* was found to be closely related to the genes encoding ANS and 3MAT1. This suggested that it may positively regulate these two genes, affecting anthocyanin accumulation and modification and thereby affecting cold tolerance. The expression of *CmNAC1* was induced by low temperature. It was closely related to genes involved in flavonoid biosynthesis in the correlation network. Simultaneously, we mined a module related to flavonoids and anthocyanins and constructed a gene co-expression network based on WGCNA. Within this network, CmNAC1 also exhibited high connectivity with genes involved in flavonoid biosynthesis. This suggested that *CmNAC1* may be induced by the low temperature and regulate flavonoid biosynthesis. Furthermore, this network also revealed potential associations between flavonoid biosynthesis genes, *UGTs*, *TFs*, and other genes. Overall, these findings have significant implications for studying Chrysanthemum cold tolerance.

## 4. Materials and Methods

### 4.1. Plant Materials and Growth Conditions

In this study, all Chrysanthemum cultivars (*Chrysanthemum morifolium*, a ground-cover Chrysanthemum cultivar) were cultivated and planted at the Flower Research Institute of the College of Life Sciences, Northeast Forestry University, Harbin, China (126°63′ E, 45°71′ N). Three Chrysanthemum cultivars, Zirong (ZR), Fenshuangdie (FSD), and Xuehaibaifan (XHBF) were selected in this study. Seedlings of all cultivars were transplanted from the field greenhouse in May, and flowering began on September 10 and continued until September 30. As previous studies have described [30], flower development was divided into six stages (S1–S6) according to the state of inflorescence development. We collected samples from the S5 and S6 stages as CK at about 22 °C on 15 September 2022. Then, the cultivars were exposed to naturally low temperatures (the average temperature is 16 °C in September and 8 °C in October; refer to Table S2 for details). The petals were collected on 14 October and 24 October and divided into three replicates in equal proportions as LT.

### 4.2. Metabolic Profiling

Metabolite detection follows the previous description [49]. In short, the petals were vacuum freeze-dried and ground (30 Hz, 1.5 min) to powder. A total of 100 mg powder was dissolved in 1.2 mL of 70% methanol extract. The dissolved samples were placed in a 4 °C refrigerator overnight and vortexed six times. Then, the samples were centrifuged at 10,000× *g* for 10 min, the supernatant was absorbed, filtered by a microporous filter membrane, and stored in a sample vial. High-performance liquid chromatography–tandem mass spectrometry (HPLC-MS/MS) was performed using an Applied Biosystems 6500 Qtrap mass spectrometer equipped with the Shim-pack UFLC Shimadzu CBM30A system (Agilent Technologies, Santa Clara, CA, USA). Analytes were separated using a Waters Acquity UPLC HSS T3 C18 column (Waters, Milford, MA, USA) (2.1 mm × 100 mm, 2.8 µm) at 40 °C. Solvent A was double-distilled water (adding 0.04% acetic acid) and solvent B was acetonitrile (adding 0.04% acetic acid). The flow rate was 0.35 mL/min and the injection volume was 2 µL. Elution gradient: 0 min, 5% B; 0–10 min, 95% B; and 11–14 min, 5% B. Mass spectrometry conditions: electrospray ionization (ESI) temperature, 550 °C; mass spectrum voltage, 5500 V; curtain gas (CUR), 30 psi; and collision-activated dissociation (CAD), high. Each ion was scanned and detected based on the decluttering potential (DP) and collision energy (CE).

### 4.3. Transcript Assay

Total RNA extraction using NEBNext^®^UltaTMRNA Library Prep Kit (Ribo-Zero kit; Epicentre, Madison, WI, USA) for Illumina^®^ was performed as described previously [50]. The mRNA was purified using magnetic beads with attached oligonucleotide (dT). The purified mRNA fragments were then broken down into small pieces with fragment buffer at the appropriate temperature. Next, the first strand of cDNA was generated through reverse transcription using random hameric primers, followed by the synthesis of the second strand of cDNA. A-TailingMix and RNA Index Adapters were added for terminal repair incubation. The cDNA fragments obtained from PCR amplification were purified using Ampure XP Beads and dissolved in EB solution. Quality control certification was performed on the Agilent Technologies 2100 bioanalyzer. The double-stranded PCR product obtained in the previous step was denatured by heating and then looped through the splint oligo sequence to format single-stranded circular DNA (ssCir DNA) into the final library.

### 4.4. WGCNA

The Weighted Gene Co-expression Network Analysis (WGCNA) was conducted using the WGCNA software package (version 1.71) in R [51]. To facilitate a more accurate acquisition of valuable gene modules, the genefilter package varFilter function uses the IQR method for filtering and retains 8456 genes for analysis. The mergeCutHeight is set to 0.25. According to the gene annotations in the module, the top 50 genes with connectivity were screened, and Cytoscape software (version 3.10.1) was used to construct the correlation network.

### 4.5. Statistical Analysis

Unsupervised principal component analysis (unsupervised PCA) was performed in R (www.r-project.org) by the statistical function prcomp. The data were scaled by unit variance before unsupervised PCA. Differentially accumulated metabolites (DAMs) were identified based on Variable Important for the Projection values (VIP > 1), corrected P-values (FDR < 0.05, Student’s *t*-test), and absolute Log_2_Fold change (|Log_2_FC| ≥ 1.0). The R-package MetaboAnalystR was utilized to calculate the VIP values of Orthogonal Partial Least Squares-Discriminant Analysis (OPLS-DA) results, including score graphs and ranking graphs [52]. Before OPLS-DA, the data were log-transformed (log_2_) and mean-centered. To prevent overfitting, a permutation test with 200 permutations was conducted. DESeq2 was used to analyze the differential expression of the two groups of genes, and the *p* value was corrected by the Benjamini and Hochberg method. The corrected *p*-values (FDR < 0.05, Student’s *t*-test) and |Log_2_FC| ≥ 1.0 were used as thresholds for significant differential expression. All heatmaps were drawn with TBtools II (version 1.12) [53].

The Kyoto Encyclopedia of Genes and Genomes (KEGG, https://www.kegg.jp/) is a comprehensive database [54]. Differentially expressed genes and differentially accumulated metabolites were analyzed using the cluster Profiler R package (version 3.4.4).

Gene annotation by gene ontology (GO) was implemented by Blast2GO software (version 2.5). Omics correlation analysis was performed using the Metware Cloud, a free online platform for data analysis (htps://cloud.metware.cn (accessed on 22 June 2024)).

### 4.6. Phylogenetic Analysis and Protein Sequence Alignment

All amino acid sequences were obtained from the National Center for Biotechnology Information (NCBI). The neighbor-joining tree was created using MEGA11 software (version 11.0.03) with all parameters as default. The reliability of the reconstructed tree was evaluated by a bootstrap test with 1000 replicates.

The protein sequence of DgbZIP3 was obtained from NCBI (MW528211.1). The protein sequences were submitted to the ClustalW website, and the Clustal algorithm was utilized for sequence comparison [55]. Subsequently, the results were submitted to ENDscript/ESPript for visualization [56].

## 5. Conclusions

Our study revealed the critical role of flavonoid metabolism in response to the low temperature in Chrysanthemums and suggested the color shift from white to purple in XHBF was caused by the specific accumulation of anthocyanins under the low temperature. Six candidate *UGTs* for flavonoid glycosylation were identified for further study. In addition, CmbZIP3 and CmNAC1 were screened out as potential transcription factors regulating cold tolerance by targeting flavonoid metabolism genes in Chrysanthemums. In conclusion, our study elucidated the pivotal role of flavonoid metabolism response to the low temperature in Chrysanthemums and offered valuable metabolic resources for future research on Chrysanthemums under low temperature.

## Data Availability

The data for this study are available upon request.

## Supplementary Materials

The following supporting information can be downloaded at: https://www.mdpi.com/article/10.3390/ijms25147589/s1.
