# Supplementary figures and images for: Integrated Metabolomics and Transcriptomics Reveal the Key Role of Flavonoids in the Cold Tolerance of Chrysanthemum
Source: Int J Mol Sci. 2024 Jul 10;25(14):7589. doi: 10.3390/ijms25147589 (PMC11276724; doi:10.3390/ijms25147589)

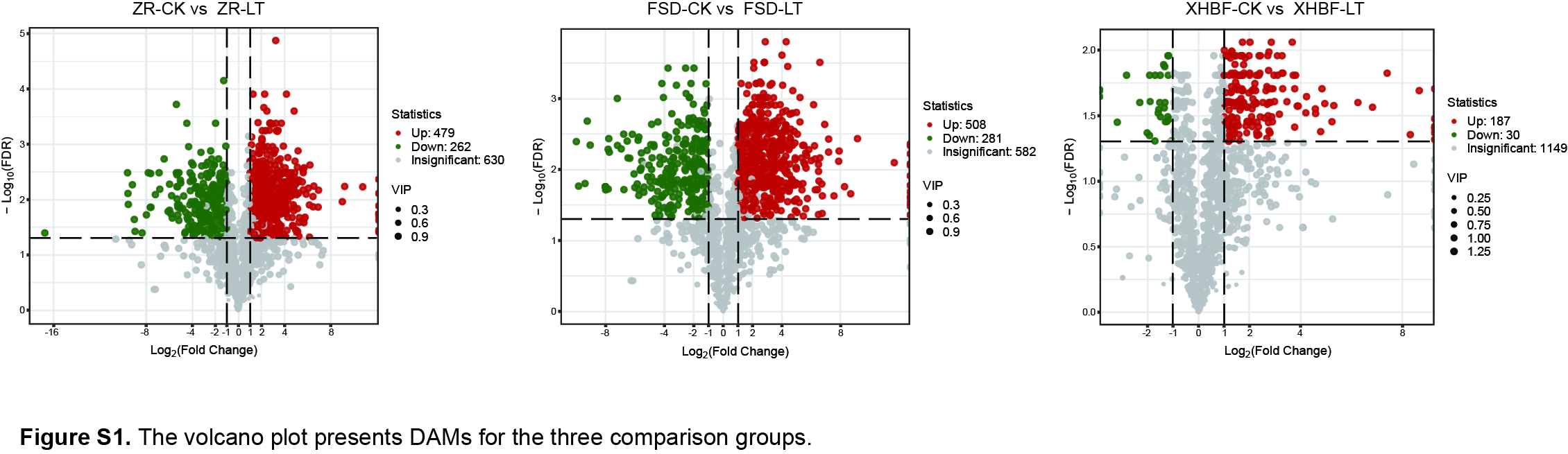

Supplement: Supplementary file 1 [file ijms-25-07589-s001.zip › Figure S1.tif]

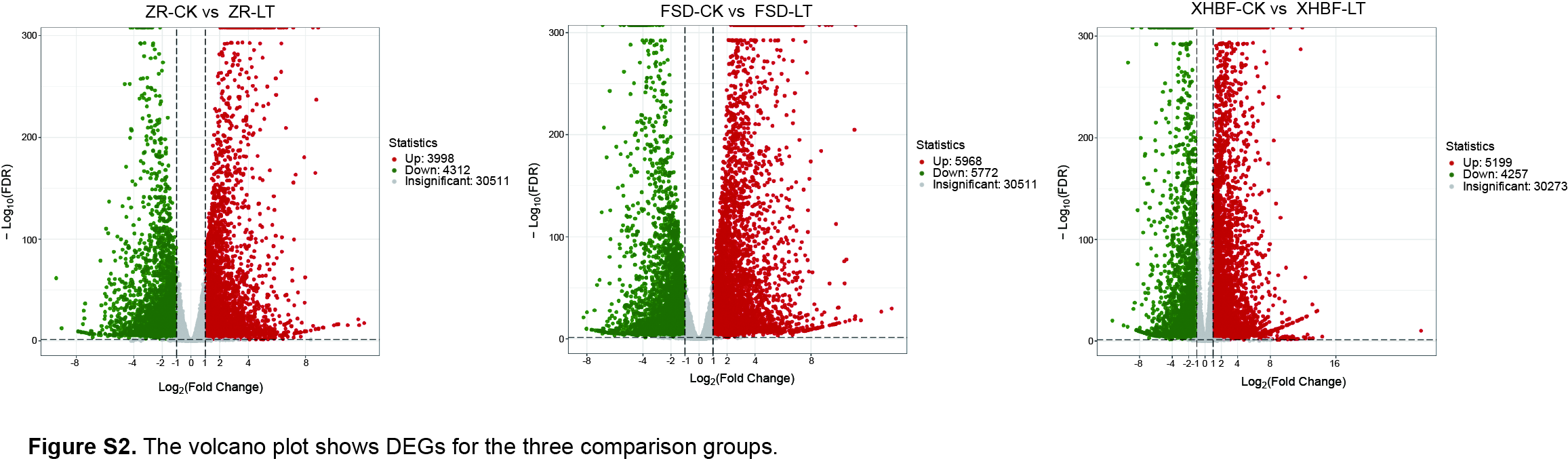

Supplement: Supplementary file 1 [file ijms-25-07589-s001.zip › Figure S2.tif]

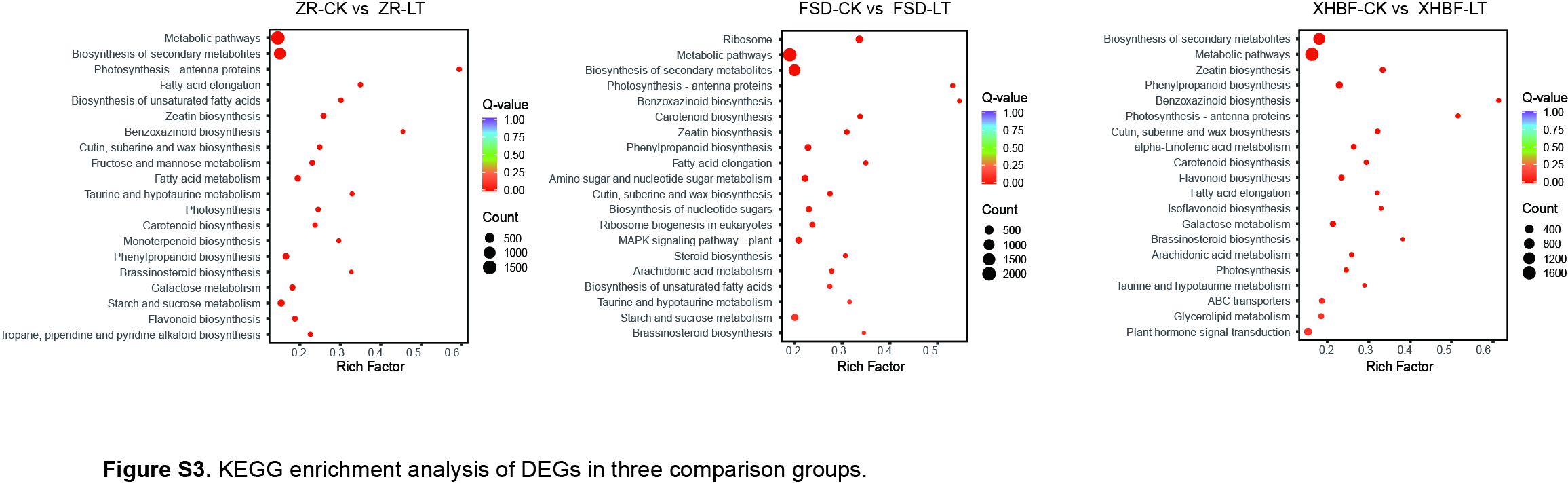

Supplement: Supplementary file 1 [file ijms-25-07589-s001.zip › Figure S3.tif]

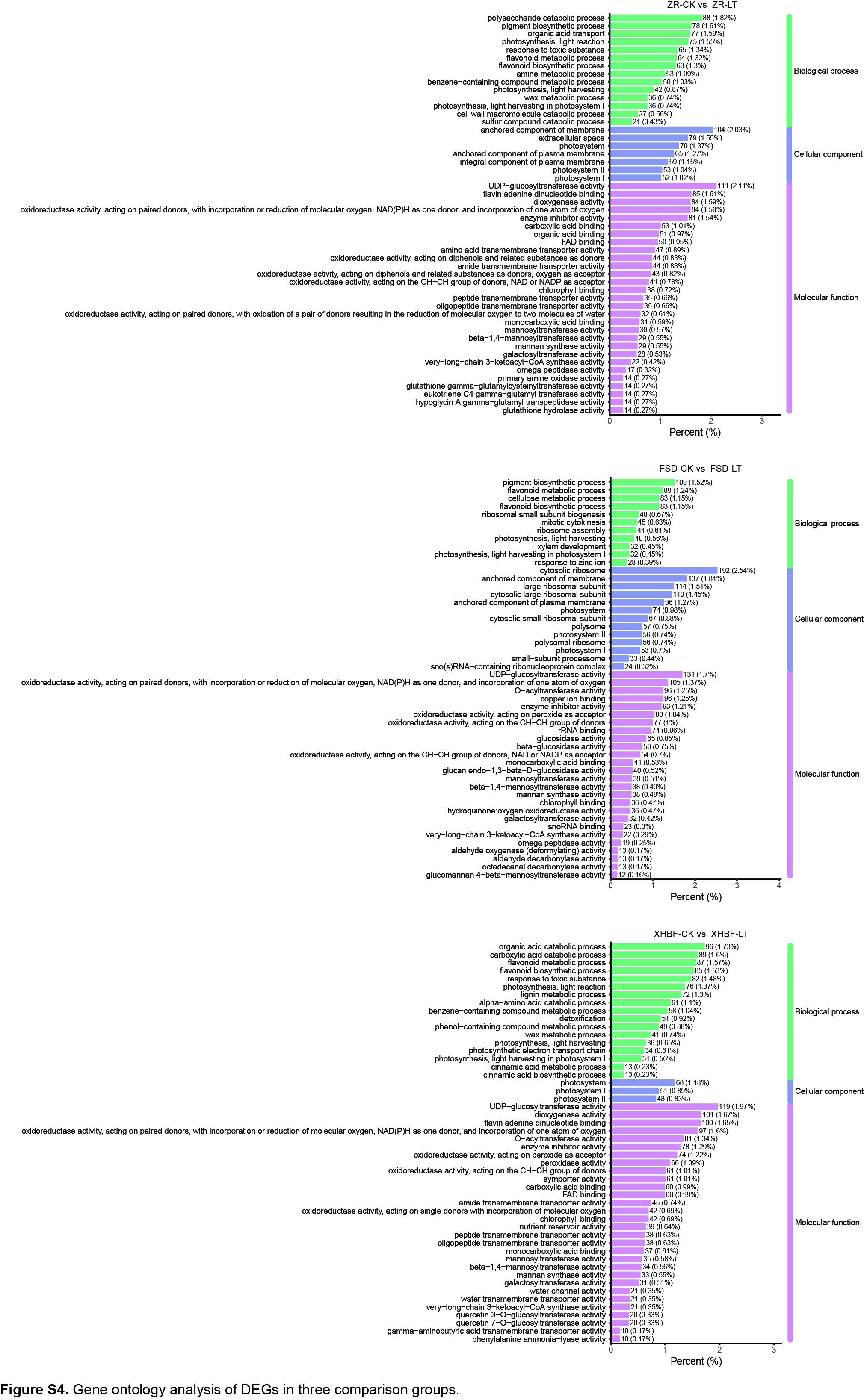

Supplement: Supplementary file 1 [file ijms-25-07589-s001.zip › Figure S4.tif]

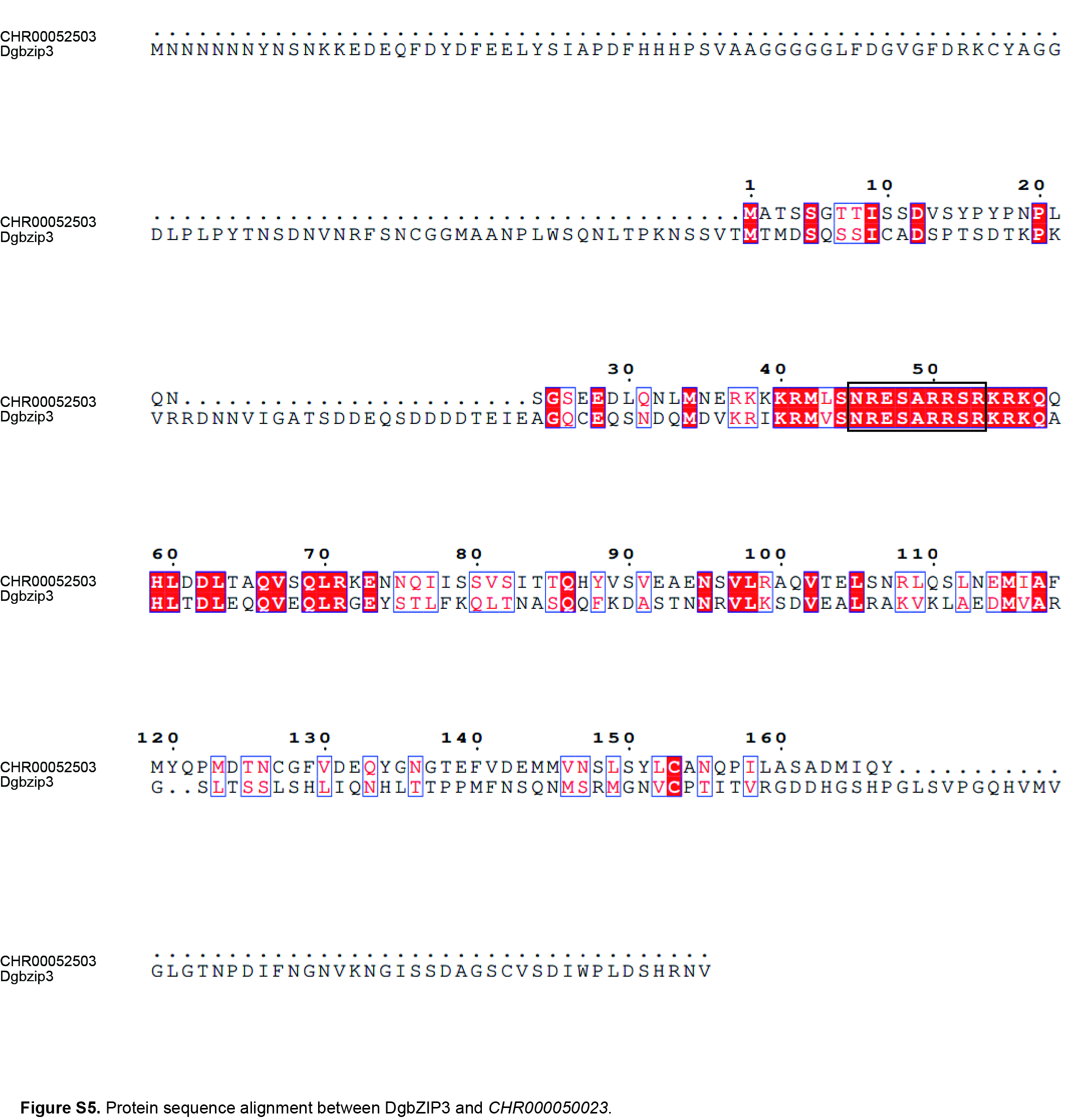

Supplement: Supplementary file 1 [file ijms-25-07589-s001.zip › Figure S5.tif]

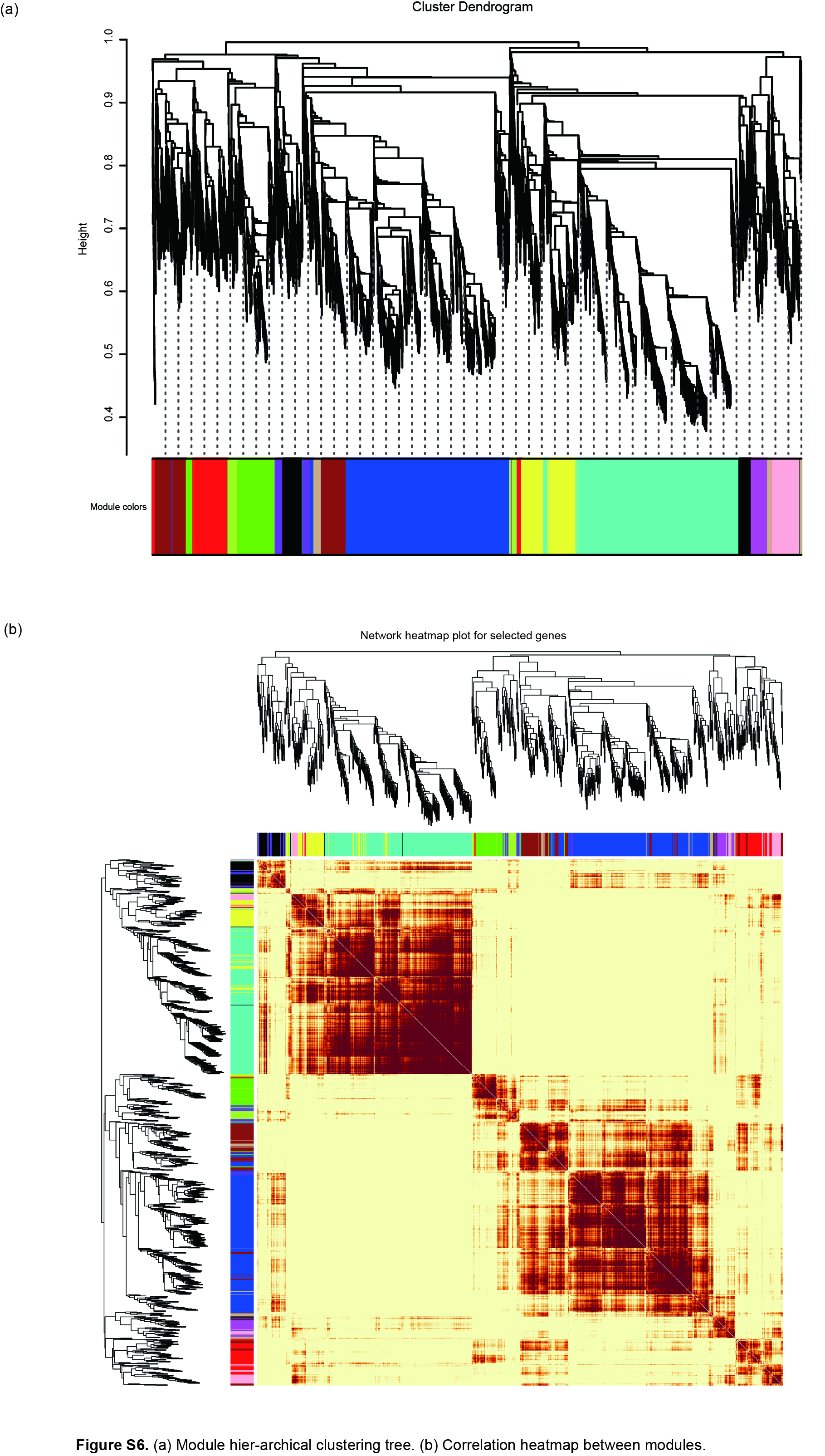

Supplement: Supplementary file 1 [file ijms-25-07589-s001.zip › Figure S6.tif]
